# 

**Published:** 1877-11

**Authors:** 


### Table of Contents.

### ORIGINAL COMMUNICATIONS.

CHRONIC AURAL CATARRH. *By C. A. Simpson, M.D., Atlanta, Ga.* . . . 449

BOTANY, IN ITS RELATIONS WITH MEDICINE—NO. 2. *By W. T. Grant, M.D., Atlanta, Ga.* . . . 454

ATLANTA MEDICAL COLLEGE CLINIC—Service of J. G. Westmoreland, M.D. *Reported by S. W. Johnson, Atlanta, Ga* . . . 457

### REPORTS OF SOCIETIES.

Atlanta Academy of Medecine . . . 460
Detroit Medical and Library Association . . . 471

### SELECTIONS.

Saccharine Diabetes . . . 475
A Contribution to our Knowledge of Phytolacca Decandra, and of Grindelia Rocusta . . . 477
Recent Progress in Otology . . . 483
On the Etiology, Pathology and Treatment of Spermatorrhœa and Impotence . . . 488
Skimmed Milk an Important Adjunct in the Treatment of Catarrh of the Bladder and Cystitis . . . 496
Bimanual or Combined Turning . . . 497

### EDITORIAL.

Medical Societies . . . 502
Obituary . . . 503
Editorial Correspondence . . . 504

### BIBLIOGRAPHICAL.

A Compend of Diagnosis in Pathological Anatomy . . . 505
Transactions of International Medical Congress of Philadelphia . . . 505
Cyclopædia of the Practice of Medicine . . . 506
Pamphlets Received . . . 506

### EXCERPTA.

Oldoini in the Treatment of Croup . . . 507
Relation of the Blood to Vaccinal Immunity . . . 507
Something New about Oxygen . . . 508
Poisoning by Santonin . . . 509
The Treatment of Tapeworm . . . 511
Death from Chloroform . . . 511
The Position of (Therapeutical) Damiana . . . 512
Nymphomania in a Mare cured by Chloral . . . 512

